# Willingness to participate in health research: Tunisian survey

**DOI:** 10.1186/s12910-016-0131-3

**Published:** 2016-08-04

**Authors:** Wahid Bouida, Mohamed Habib Grissa, Asma Zorgati, Kaouthar Beltaief, Hamdi Boubaker, Asma Sriha, Riadh Boukef, Semir Nouira

**Affiliations:** 1 Emergency Department, Fattouma Bourguiba University Hospital Monastir, Monastir, 5000 Tunisia; 2 Emergency Department, Sahloul University Hospital, Sousse, Tunisia; 3 Community Medicine Department, Fattouma Bourguiba University Hospital Monastir, Monastir, Tunisia; 4 Research Laboratory (LR12SP18), University of Monastir, Monastir, Tunisia

**Keywords:** Informed consent, Patient participation, Muslim community medical research

## Abstract

**Background:**

Few studies have identified the willingness rate of developing countries population to be enrolled in clinical trials.

**Methods:**

All participants including patients (*n* = 612), healthy volunteers (*n* = 354) and doctors (*n* = 134) completed a questionnaire to examine factors affecting the consent to participate in medical research.

**Results:**

Overall, 80 % of the included population agree to participate in health research. This rate was lower for trials dealing with life-threatening diseases (38 %). Altruism and perceived risk of harm were the main reason to respectively accept or refuse to participate in clinical trials. Factors significantly associated with willingness were: age <40 years (Odds Ratio (OR) 1.6 [95 % Confidence Interval (CI) 1.2-2.1]) and prior history of blood donation (OR 2.4 [95 % CI 1.7-3.5]).

**Conclusion:**

Most participants expressed their willingness to be included in medical research especially if they are young or if they have history of blood donation. However, consent to participate is low when medical research required acute care.

**Electronic supplementary material:**

The online version of this article (doi:10.1186/s12910-016-0131-3) contains supplementary material, which is available to authorized users.

## Background

In the past decade, there is a growing trend in designing big trials that integrate many thousands of persons with possibility of using different data sources [1–3]. However, many observers noted the underrepresentation of patients from developing countries in these trials, especially Muslim communities which raises concerns about the generalizability of their results [4, 5]. Socio-cultural factors like illiteracy, logistical barriers, and distrust in clinical research may influence people’s decision to participate in biomedical research and possibly limit the rate of consent [6–9]. To our knowledge, there is no study that assessed the willingness of Muslim and Arab communities to participate in individual research trials. The purpose of our study was to evaluate willingness to participate in medical research in a Tunisian population, and determine factors that could influence their consent.

## Methods

This is a transversal survey performed in the outpatient clinic of three Tunisian university hospitals from November 2010 to February 2011. Participants were screened for inclusion on the basis of convenience sampling. Potential participants in our survey were recruited either as patients waiting for their clinic appointments or as healthy volunteers accompanying their relatives. Both medical and surgical patients were recruited. Most medical patients were from cardiology, endocrinology and rheumatology clinics. We also included a sample of doctors working in the participating hospitals. Most of them (92 %) are specialist senior in their disciplines (emergency department, cardiology, pneumology, anesthesiology, internal medicine, surgery, pediatry…). We used a face-to-face questionnaire (Additional file 1) and the answers were recorded by one of the investigators who gave information about the survey and the questionnaire. Exclusion criteria were acutely ill patients, persons under 18 years, persons who choose not or unable to participate and those who came to the hospital during the weekend.

The survey tool consisted of open-ended questions conducted in Arabic language. The questionnaire assessed the attitudes of the individuals regarding some items: willingness to participate in clinical trials, reason for acceptance, reasons for refusal, attitude towards the enrollment of a very sick relative in medical research, and opinion about investigator’s main goals behind doing research.

For all participants included in the study, demographic data were collected and stored on a standard clinical record form. These included age, sex, marital status, educational level, morbidity, prior participation in a medical research and history of blood donation. The questionnaire was anonymous.

### Data analysis

Continuous variables were expressed as means and standard deviations when they were normally distributed and as medians (95 % confidence interval) when they were not normally distributed. Continuous variables were compared using one way ANOVA Test or Kruskal-Wallis test depending on the validity conditions of each test. Categorical variables were compared using the chi-squared test or Fisher’s exact test, as appropriate. To examine the factors that possibly influence participant willingness, a multiple logistic regression analysis was performed. Acceptance to participate was the dependent variable and the independent variables were included in the model at the risk of 20 %. The difference is considered statistically significant only for values of *p* ≤ 0.05. The data obtained in this study were collected, stored and analyzed using SPSS (version 18.00).

## Results

**Fig. 1:**
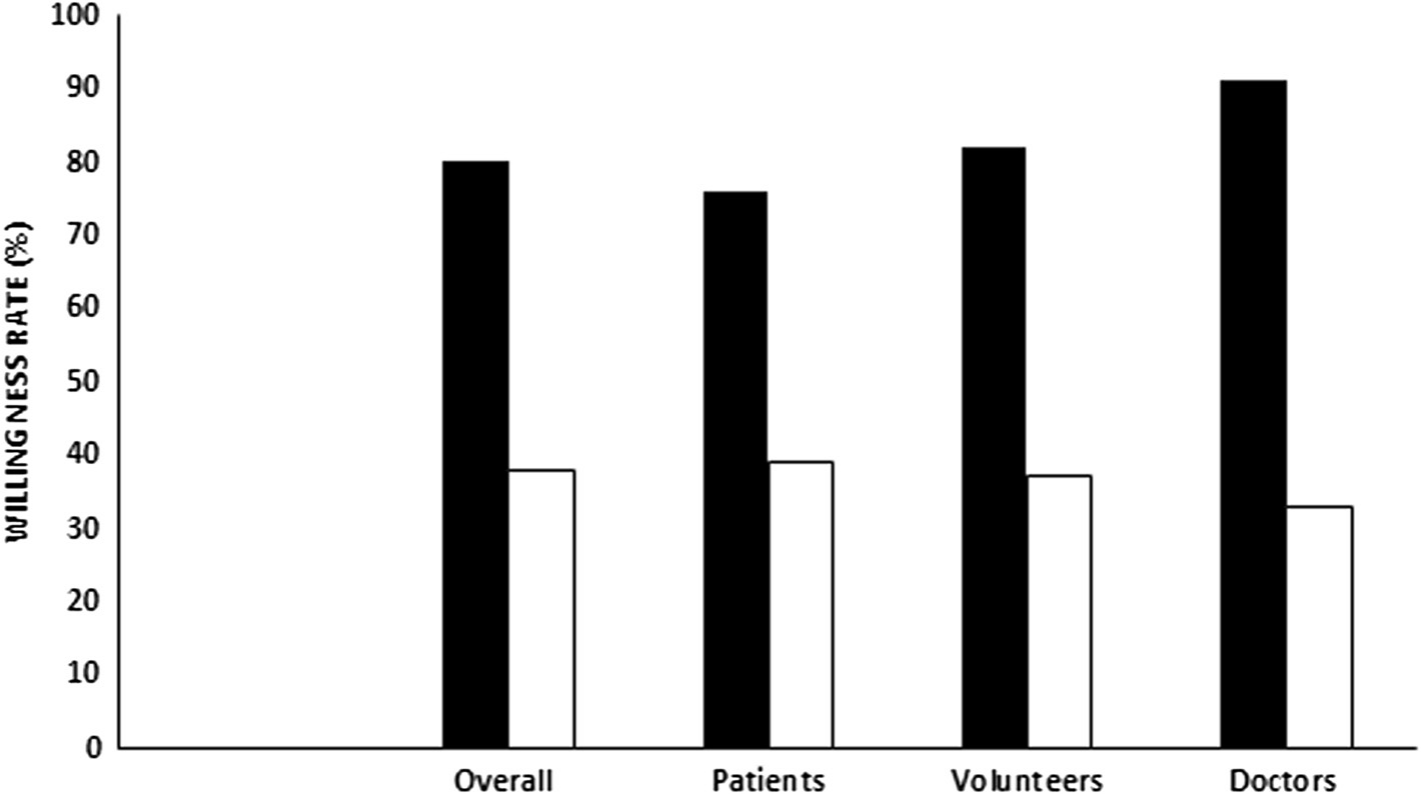
Rate of willingness to participate in medical research (solid bars) and research dealing with critically ill patients (open bars). No statistically significant difference between groups

**Fig. 2:**
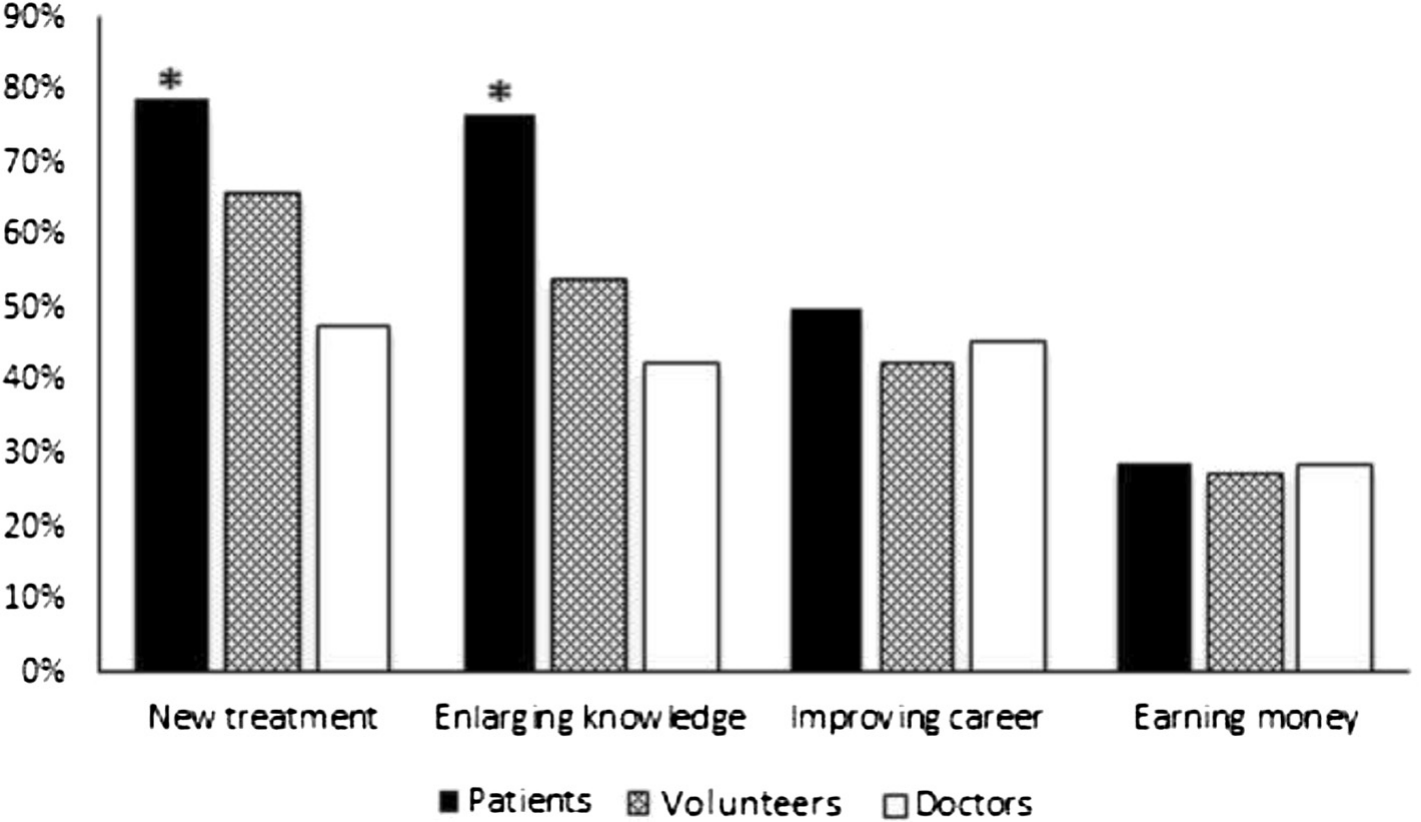
Main objectives of medical researchers from the point of view of patients, volunteers and doctors. *p < 0.05 between patients vs volunteers and doctors

During the study period, 1201 participants were included and 1100 completed the questionnaire (completion rate 91.6 %). We included 612 in patients group (56 %), 354 in healthy volunteers group (32 %) and 134 in doctors’ group (12 %). Overall, 676 (61 %) were younger than 40 years, 21 % had no co-morbidities, and 34.5 % had a previous history of blood donation. Demographic of study participants characteristics are described in Table 1. Mean age was lowest in volunteers group while patients group had the highest proportion of married and the lowest rate of high education level. Doctors group had the highest proportion of history of prior participation in medical research and blood donation. Our results reported 80 % overall rate of willingness to participate in medical research but this rate was lower when the research concerns a life-threatening situation (38 %). Rate of willingness was significantly higher in doctors’ group compared to patients and volunteers (Fig. 1). Altruism and contribution to health care improvement were the common reasons for participation acceptance. In fact, 40 % of patients accept to be enrolled in a medical research bearing in mind the importance of taking part in health care improvement. Perceived risk of harm was the major reason for refusal (66 %) and only 6 % of participants think that medical research are useless (Table 2). Healthy volunteers, young participants (<40 years), high education level, presence of chronic disease and prior history of blood donation were associated with willingness to participate in a medical research (Table 3). The independent factors were young participant (age < 40 years) and history of blood donation (OR 1.6 [95 % CI 1.2-2.1] and 2.4 [95 % CI 1.7-3.5] respectively). There was a general positive opinion about the intention of the investigators leading studies. The majority of respondents in particular patients group believe in the aim of finding new treatments (70.6 %), and enlarging knowledge with medical research (65.2 %). Difference between patients and the two other groups was significant (*p* = 0.001). Improving investigator’s career and earning money were considered less important as motivation to conduct medical research (Fig. 2).

**Table 1:** Demographic characteristics of the three participating groups

Variables	Patients (*n* = 612)	Volunteers (*n* = 354)	Doctors (*n* = 134)
Age mean years (SD)	42.3 (15.8)	25.9 (9.8)*	35.6 (9.5)
Sex Male n (%)	196 (32)	131 (37)	60 (45)**
Married n (%)	447 (73)	123 (29)*	86 (64)
History of chronic disease n (%)	213 (35)	42 (12)	22 (16)
arterial hypertension	105	19	9
diabetes	72	18	10
coronary heart disease	51	6	2
chronic obstructive pulmonary disease	24	0	0
rheumatic disease	18	9	1
neoplasia	10	0	0
others	5	11	7
High school education n (%)	318 (52)	244 (69)	134 (100)**
Prior participation in medical research n (%)	24 (4)	28 (8)	32 (24)**
History of previous blood donation n (%)	184 (30)	124 (35)	68 (51)**

*p < 0.05 vs patients and doctors groups. **p < 0.05 vs patients and volunteers groups

**Table 2:** Reasons for acceptance or refusal to participate in medical research for the overall population

	n (%)
Reasons for acceptance *n* = 891	
Altruism	356 (33.3)
Health care improvement	356 (33.3)
Help patients	44 (4.9)
Other reasons	153 (28.5)
Reasons for refusal *n* = 209	
Risk of harmful effects	138 (66.0)
Distrust	44 (21.0)
Researchs are useless	12 (5.7)
Others reasons	15 (7.3)

**Table 3:** Comparison between survey participants who accept and who do not accept to participate in medical research

	Accept to participate		
	Yes *n* = 891	No *n* = 209	*p*
Participants n (%)			
Patients	488 (79.7)	122 (20.31)	0.34
Volunteers	281 (79.3)	73 (20.7)	
Doctors	122 (91)	12 (9)	
Age < 40 years n (%)	557 (62.5)	109 (52)	0.05
High school education n (%)	622 (70)	80 (38)	<0.01
Sex male n (%)	320 (36)	74 (35)	0.89
Not married n (%)	335 (37)	80 (38)	0.85
History of chronic disease n (%)	277 (31)	21 (10)	<0.01
Prior participation in medical research n (%)	75 (8)	6 (3)	<0.01
History of blood donation n (%)	335 (37)	46 (22)	<0.01

## Discussion

The main result of our study was the positive attitude toward medical research in our population with a high rate (80 %) of willingness to participate especially in medical doctors group (89 %). Altruism and the desire to contribute to medical care improvement were the main motivation for participation while risk of harmful effects and distrust were the main reason for refusal. The independent factors associated with willingness were age <40 years and history of blood donation.

The progress of medicine today is a result of biomedical research which necessarily involves human participants. The adequate representation of racial and ethnic minorities in clinical trials is required to reach meaningful results. This issue is of great importance for the relevance of international trials involving many countries. However, most research participants in available trials continue to be recruited from USA and Europe while those from developing countries especially from Muslim communities continue to be underrepresented [4]. Differences related to socioeconomic status, ethnicity, and education may constitute a limit to their participation. In areas with low literacy level and population unfamiliar with research concepts recruiting subjects to medical trials is a big challenge. Unexpectedly, we found a high rate of willingness in our study population. This could be related to the trust of our population towards their doctors. Experience has shown that paternalism model where doctors’ opinions are accorded a larger role in decision-making is an accepted pattern of informed consent in such settings. Our results were similar to many studies performed in developed countries such America, Denmark, England, Belgium and France [4, 10–12]. Similarly, in a recent study conducted in Saudi Arabia, it was demonstrated that a significant number of oncology patients (61 %) were willing to participate in clinical trials [13]. This means that in Arab and Muslim regions patient’s awareness and perception of medical research are not limiting factor in clinical trials recruitment. In contrast, a low rate of consent was reported in Japanese population [14] which means that ethnicity and race are two unmodified factors that may influence attitude to research. The same findings were observed with African and Latin Americans where the lack of participation is partly explained by the level of suspicion and mistrust among minority communities regarding their participation in medical research [5, 15, 16]. It should be highlighted that consent to participate to medical research depends also on individual factors. For example, more educated patients may have a better understanding of the social benefits of clinical research. Available studies showed that middle-aged patients and those with a favorable experience of health care may be more willing to participate [17]. In our study, we found that young age, high school education level, and prior history of blood donation were associated with better acceptance of taking part to biomedical research. Altruism was a common reason to accept participation to medical research in our population. In previous studies [11, 18, 19] receiving personal benefits like better treatment [20] was also highlighted. The main reason for refusal to participate in medical research in our study was the fear of side effects; this concern was found in other surveys [21]. Obviously, none would participate in research activities unless they feel safe and are treated with dignity and trust [13, 15, 22, 23]. Factors related to research methodology could also influence the response attitude. It was demonstrated that randomization and blinding can cause anxiety, discomfort, and confusion among participants [21]. This is also common in medical emergency situations and can cause a significant impediment to the conduct of clinical trials including critically ill patients. The vulnerable nature of critically ill patients raises issues of patient safety, and consent to participate in clinical trial could be difficult. Our results support this feeling in demonstrating fear of our population from the potential incremental risk posed by participation in intensive care research. Would different results have been obtained if the survey had been conducted under real life conditions? The answer to this question is not obvious with regard to the nature and complexity of urgent decision in presence of devastating illness that makes objective evaluation of the informed consent process difficult. Whatever the willingness rate in this setting, it is essential that the health care professionals recruiting research participants are well trained in order to provide comprehensive information and obtain the required trust. Researchers trust is an important factor in willingness process and may explain the high rate observed in our study. In support to these findings the positive believe at our participants with regard to the main objective of investigators in conducting research.

There were limitations to this study. First, our study assessed the attitude toward medical research in general and so, the participants can feel themselves less concerned and probably more enthusiastic to participate than they are really. Their opinions were an intention more than an effective decision. Second, our sample was limited to those who agreed to participate in this study and probably it represents who are more willing to participate. Third, our study focused on overall clinical trial participation. Our search did not specify whether the willingness could be different in observational versus interventional study. Fourth, in this survey we assessed only the participant point of view. As paternalism and coercion are current model of medical care in our cultural behavior, we need to evaluate the practices among attending professionals in matters relating to informed consent to assess objectively its validity when obtained in such conditions. Fifth, our survey was not designed to assess willingness to participate in medical research including children. This raises specific ethical complexities that are not under the scope of this study.

## Conclusions

Most of the participants in this survey indicated that they were willing to take part in clinical research in particular doctors and young people. Our findings are an important step in remedying any underrepresentation of Muslim groups in international clinical trials.

## Abbreviations

CI, confidence interval; OR, odds ratio

## Additional file

Additional file 1: Questionnaire. It was anonymous, face-to-face questionnaire and the answers were recorded by one of the investigators who gave information about the survey. (DOCX 23 kb)
