# Supplementary material for: Willingness to participate in health research: Tunisian survey
Source: BMC Med Ethics. 2016 Aug 4;17:47. doi: 10.1186/s12910-016-0131-3 (PMC4973371; doi:10.1186/s12910-016-0131-3)
Supplement: Additional file 1: — Questionnaire. It was anonymous, face-to-face questionnaire and the answers were recorded by one of the investigators who gave information about the survey. (DOCX 23 kb) [file 12910_2016_131_MOESM1_ESM.docx]

**Questionnaire**

**Demographics:**

**Age:**

**Sex:**

**Schooling:**

**Area of residence:**

**Question 1.** Did you previously participate in a medical research as a volunteer or as patient?

Yes

No

**Question 2.** How is your attitude to your own potential participation in a medical research as a volunteer or as a patient?

Would accept

Would not accept

Do not know

**Question 3.** If you accept to participate in a medical research, what are the reasons?

Desire to help other people

Health care improvement

Fear that doctors would not provide good care

Other reasons

**Question 4.** If you refuse to participate in a medical research, what are the reasons?

Risk of harmful effects

Distrust towards researchers

Researchs are useless

Other reasons

**Question 5.** What do you think is the purpose of performing a medical research? You may choose more than one answer

To improve care of future patients

To increase doctors knowledge in general

To improve care of the patient in the research

So that drug companies can earn more money

**Question 6.** What would be important if you participate in a medical research? You may choose more than one answer.

That you gain directly from the research

That future patients will gain from the trial

That new knowledge is accomplished to develop new therapies

That there is no risk by participation

**Question 7.** How is your attitude if your relative, who is in intensive care, was to participate in a medical research?

Would accept

Would not accept

Do not know

**Question 8.** If you don’t accept, why?

Fear from potential risk

Discomfort for your relative

Not accepted ethically

Other

**Question 9.** What motives do you think doctors have when they perform medical research? You may choose more than one answer.

Wish to find new treatments

Wish to gain new knowledge

Wish to improve career

**Question 10.** Have you any previous chronic disease?

Arterial hypertension Yes No

Diabetes Yes No

Coronary heart disease Yes No

Chronic obstructive pulmonary disease Yes No

Chronic inflammatory disease Yes No

Others Yes No

**Question 11.** Did you participate in blood donation?

Yes No
